# Late presentation for diagnosis of HIV infection among HIV positive patients in South Tigray Zone, Ethiopia

**DOI:** 10.1186/s12889-016-3263-y

**Published:** 2016-07-12

**Authors:** Admassu Assen, Fantahun Molla, Abrham Wondimu, Solomon Abrha, Wondim Melkam, Ebisa Tadesse, Zewdu Yilma, Tadele Eticha, Hagos Abrha, Birhanu Demeke Workneh

**Affiliations:** Department of Pharmacy, College of Health Sciences, Mekelle University, Mekelle, Ethiopia; Department of Internal Medicine, College of Health Sciences, Mekelle University, Mekelle, Ethiopia

**Keywords:** Late HIV diagnosis, Positive, HIV infection, Tigray, Ethiopia

## Abstract

**Background:**

In spite of the availability and accessibility of HIV testing opportunities and efforts, people are being late to test in the course of HIV infection. Late diagnosis leads to late anti-retroviral therapy initiation which in turn results in poor treatment outcome and prognosis of the disease. The aim of this study was to determine the prevalence and predictors of late HIV diagnosis among HIV-infected patients in South Tigray Zone, Ethiopia.

**Methods:**

A facility based cross sectional study was conducted among HIV positive patients from February 1-30, 2014 in Southern Tigray, Ethiopia. Multistage sampling technique was employed to select the study participants. Data were collected by reviewing patient medical card and interviewing using structured questionnaire. Data were entered using Epi-Data version 3.1 and analyzed using SPSS version 20.0. Both bivariate and multivariate logistic regressions were modeled to evaluate the association of predictors with late diagnosis of HIV infection.

**Results:**

Out of 789 study participants, 68.8 % of them were late for HIV diagnosis. Feeling healthy (65.7 %), fear of stigma and discrimination (32.4 %) and using traditional treatment (1.5 %) were reported as the main reasons for late HIV diagnosis. Use of Khat [AOR = 3.27, 95 % CI (1.75, 6.13)], bed ridden functional status [AOR = 2.66, 95 % CI (1.60, 4.42)], ambulatory functional status [AOR = 1.56, 95 % CI (1.03, 2.35)] and Muslim religion [AOR = 2.26, 95 % CI (1.13, 4.49)] were significantly associated with late presentation for HIV diagnosis.

**Conclusions:**

High prevalence of late HIV diagnosis was recorded in Southern Tigray Zone, Ethiopia. Public health educations and campaigns targeted at improving early diagnosis and prognosis of people living with HIV/AIDS in Southern Tigray, Northern Ethiopia should be underway.

## Background

The natural history of HIV disease is characterized by an asymptomatic stage that progress to clinical complications. The clinically latent period, in spite of variable duration, often lasts for years in an infected person. Before clinical presentation, whether an HIV antibody test is performed or not, the latent period depends on a variety of factors. As a consequence, time lag between HIV infection and diagnosis differs greatly among those infected patients [1].

Three decades have passed since HIV testing first became available. Despite the availability and accessibility of HIV testing, people continue to test late in the course of HIV infection [2, 3]. Testing, diagnosis and medication care soon after HIV infection and before developing opportunistic infections and other AIDS defining illness and clinical AIDS, can prevent illness, improve survival and reduce transmission [4]. On the other hand, patients receiving HIV diagnosis late in the course of infection are usually severely immunocompromised, more likely to present with co-morbidities like tuberculosis, and have short term mortality. Delay in diagnosis adversely impact both disease prognosis at patient level and transmission at community level [5]. An early diagnosis provides opportunity of reducing or halting further transmission due to change in risk behavior. Besides, due to a higher viral burden in these patients, the likelihood of transmission from these patients is also very high compared to individuals diagnosed early in the course of infection [5, 6]. Being too late for HIV testing is one of the reasons for delayed access to antiretroviral therapy for people in need. High mortality in the months after initiation of the treatment was reported in some studies conducted in Sub Saharan Africa.

Several factors were found to be associated with late diagnosis such as being male, working outside home, perceived health delivery barriers, receiving health care from non-medical providers, having poor emotional health, living far from the clinic, not knowing primary partner’s HIV status and having had a gynecological pathology in the last year [7–9]. However, there was no a single previous study in the study area that tried to investigate the magnitude of late HIV diagnosis and its associated factors. Hence, the objective of this study was to examine the prevalence and predictors of late HIV diagnosis among HIV-positive patients in Southern Tigray, Ethiopia.

## Methods

### Study area and design

Facility-based cross sectional study was conducted among HIV positive patients who had been attending HIV/AIDS care and treatment in the selected health centers and hospital in South Tigray Zone from February 1- 30, 2014. Southern Tigray is one of the seven administrative zones of Tigray Regional state. Tigray region forms the northernmost reaches of Ethiopia and its capital is Mekelle, which is located 783 km away from Addis Ababa. Based on the 2007 Census conducted by the Central Statistical Agency of Ethiopia, the region has an estimated total population of 4,314,456 and 985,654 households with averagely 4.4 persons per household.

### Sample size determination and sampling procedure

A single population proportion formula was used to determine the sample size. The prevalence of late HIV diagnosis was taken as 50 % to get maximum sample size since no prior study was conducted in the study area. Then, after considering 5 % margin of error at 95 % confidence level, 5 % non-response rate and design effect of 2, the calculated sample size was 805.

Multistage sampling technique was employed to select the study participants. In the study area there are 35 health facilities. In first stage, the health facilities were stratified in to hospitals (4) and health centers (31). In stage II, to ensure representativeness 20 % of each stratum (Health center and Hospital) was included in the study. Accordingly, six health centers (Adishehu, Betemera, Korem, kukuftu, Alamata, Timuga) and one hospital (Michew Hospital) were selected by simple random sampling. In stage III, the total sample was proportionally allocated to each study site based on their patient load.

### Data collection and analysis

Data were collected using patient medical card review and structured interview. Independent and dependent variables that were used to assess the late presentation of participants to HIV diagnosis were determine by retrospectively reviewing patient cards using data abstraction format. The patient cards were selected by lottery method from the appointment list. HIV infected patients, whose card was reviewed, were also interviewed using structured questionnaire to measure magnitude of late HIV diagnosis and associated factors. The interview made after sufficient information about the study was given and got consent from the participants. During data collection one patient refused to participate and 15 other participant’s data was excluded from analysis because of incompleteness.

Patients were considered as late presenters for diagnosis of HIV infection if they were placed on anti-retroviral therapy in less than 3 months of their first positive HIV test [10–13]. Data were coded, checked for completeness and consistency. Epi-Data version 3.1 and SPSS version 20.0 statistical software were used to enter and analyze the data. Frequency and percentage distribution of variables were done in order to describe them in relation with the study population. Odds ratio (OR) was used to assess association between dependent and independent variables. Variables which showed a statistically significant association (*P* < 0.05) were analyzed at multivariate level by means of a logistic regression.

### Ethical considerations

Ethical approval and clearance were obtained from Mekelle University, College of Health Sciences Ethical Review Committee. At all levels, officials were contacted and permission was secured. The objectives of the study were explained to the study participants prior to data collection, and their written consents were sought and secured, confidentiality was ensured and the questionnaires were filled only by those who agreed to participate in the study.

## Results

### Patient characteristics

Of the total of 805 participants, 789 participants were interviewed and involved in the study which gives the response rate of 98.01 %. The rest 16 participants were excluded from the study due to incomplete information on their medication record (15 participants) and critical illness (1 participant). Nearly two third (63 %) of the participants were females and 37 % were males. Most of the study participants (37.8 %) were in age group of 31-40. Majority of the participants were Orthodox Christians (87.6 %), married (49.9 %), illiterate (60.9 %) and unemployed (73.5 %) (Table 1).

**Table 1:** Socio-demographic characteristics of the study participants in Southern Tigray, February, 2014

Variables	*N* =789 (%)
Sex	
Male	292 (37)
Female	497 (63)
Age (years)	
< 20	36 (4.6)
21–30	281 (35.6)
31–40	298 (37.8)
41–50	119 (15.0)
> 51	55 (7.0)
Religion	
Orthodox	691 (87.6)
Muslim	93 (11.8)
Protestant	4 (0.5)
Catholic	1 (0.1)
Marital status	
Single	76 ( 9.6)
Married/In union	394 (49.9)
Widowed	87 (11.0)
Divorced	232 (29.5)
Educational status	
Illiterate	481 (60.9)
Primary school	202 (25.6)
Secondary school	74 (9.4)
Tertiary	32 (4.1)
Employment status	
Employed	209 (26.5)
Unemployed	580 (73.5)

### Prevalence of late HIV diagnosis

Among the study participants, 68.8 % of them were late for HIV diagnosis. The reasons reported by the participants for being late for HIV test were feeling healthy 357 (65.7 %), fear of stigma and discrimination 176 (32.4 %), using traditional treatment 8 (1.5 %) and two participants mentioned other reasons.

### HIV risk perception and reasons for HIV test

Perception of risk of HIV-infection and reasons for HIV test is presented in Table 2. Before HIV test, two third of the patients (66 %) did not feel as they were at risk of HIV. One third of the patients felt the risk of HIV infection for the major reasons of having unprotected sex (34.7 %) and unfaithful partner (28.4 %). The majority of the patients (44.2 %) underwent diagnosis following illness while about a third (35.6 %) to know their status.

**Table 2:** HIV/AIDS risk awareness, reported reasons for risk feeling and test in Southern Tigray, February, 2014

Variables	Total	Late diagnosed	Note–late diagnosed
	*N* (%)	*n* (%)	*n* (%)
Feeling at risk for HIV			
NO	521 (66.0)	354 (65.2)	167 (67.9)
YES	268 (34.0)	189 (34.8)	79 (32.1)
Reason for feeling at risk for HIV infection			
Having unprotected sex	93 (34.7)	71 (37.6)	22 (27.8)
Having multiple sexual partner	53 (19.8)	34 (18.0)	19 (24.1)
Having unfaithful partner	76 (28.4)	52 (27.5)	24 (30.4)
Having HIV positive partner	28 (10.4)	20 (10.6)	8 (10.1)
Other	18 (6.7)	12 (6.3)	6 (7.6)
Reason for HIV testing			
To know one’s status	281(35.6)	184 (33.9)	97 (39.4)
Following illness	348 (44.2)	265 (48.8)	83 (33.7)
During a campaign	9 (1.1)	2 (0.4)	7 ( 2.8)
Antenatal follow up of the partner	60 (7.6)	30 (5.5)	30 (12.2)
Following illness to know my status	60 (7.6)	48 (8.8)	12 (4.9)
Other	31 (3.9)	14 (2.6)	17 (7.0)

### Factors associated with late HIV diagnosis

Results of logistic regression analysis are shown in Table 3. In bivariate analysis, the associations between different types of variables and being late for HIV diagnosis were statistically significant. However, these associations failed to maintain their statistical significance in multivariate analysis except for the following variables: use of Khat [AOR = 3.27, 95 % CI (1.75, 6.13)], ambulatory functional status [AOR = 1.56, 95 % CI (1.03, 2.35)] and bed ridden functional status [AOR = 2.66, 95 % CI (1.60, 4.42)] of patients and Muslim religion [AOR = 2.26 95 % CI (1.13, 4.49)] which sustained their strong association with increased odds of being late.

**Table 3:** Association of different covariates and late for HIV diagnosis among HIV positive patients in Southern Tigray, February, 2014

Characteristics	Late HIV diagnosis		Crude OR (95 % Cl)	Adjusted OR (95 % Cl)
	Yes n (%)	No n (%)		
Sex				
Male	222 (40.9)	70 (28.5)	1	1
Female	321 (59.1)	176 (71.5)	1.74 (1.26, 2.41)	1.41 (0.91,2.18)
Age (years)				
< 20	18 (3.3)	18 (7.3)	1	1
21–30	180 (33.1)	101 (41.1)	1.78 (0.89,3.58)	0.55 (0.20,1.51)
31–40	225 (41.4)	73 (29.7)	**3.08 (1.52,6.24)**	0.94 (0.48,1.86)
41–50	86 (15.8)	33 (13.4)	**2.61 (1.21,5.61)**	1.44 (0.74,2.78)
> 51	34 (6.3)	21 (8.5)	1.62 (0.69,3.79)	1.23 (0.60,2.55)
Religion				
Orthodox	458 (84.3)	233 (94.7)	1	1
Muslim	81 (14.9)	12 (4.9)	**3.43 (1.84,6.43)**	**2.26 (1.13,4.49)**
Protestant	3 (0.6)	1 (0.4)	1.53 (0.16,14.75)	1.15 (0.11,12.14)
Catholic	1 (0.2)	0	8.2188	5.7868
Marital status				
Single	52 (9.6)	24 (9.8)	1	1
Married/In union	272 (50.1)	122 (49.6)	0.97 (0.57,1.65)	0.68 (0.30,1.54)
Widowed	61 (11.2)	26 (10.6)	0.96 (0.68,1.36)	1.08 (0.57,2.03)
Divorced	158 (29.1)	74 (30.1)	1.05 (0.63,1.75)	1.09 (0.51,2.33)
Educational status				
Illiterate	314 (57.8)	167 (67.9)	0.63 (0.28,1.43)	0.5 (0.19,1.32)
Primary	155 (28.5)	47 (19.1)	1.10 (0.46,2.61)	0.94 (0.35,2.53)
Secondary	50 (9.2)	24 (9.8)	0.69 (0.27,1.77)	0.61 (0.21,1.74)
Tertiary	24 (4.4)	8 (3.3)	1	1
Employment status				
Employed	144 (26.5)	65 (26.4)	1.01 (0.71,1.41)	0.72 (0.48,1.10)
Unemployed	399 (73.5)	181 (73.6)	1	1
Opportunistic infection				
No	247 (45.5)	129 (52.4)	1	1
Yes	296 (54.5)	117 (47.6)	1.32 (0.98,1.79)	0.97 (0.66,1.41)
Functional status at presentation				
Ambulatory	172 (31.7)	65 (26.4)	**1.76 (1.24,2.50)**	**1.56 (1.03,2.35)**
Bedridden	142 (26.2)	29 (11.8)	**3.25 (2.08,5.09)**	**2.66 (1.60,4.42)**
Working	229 (42.2)	152 (61.8)	1	1
Khat Chewing				
No	465 (85.6)	234 (95.1)	1	1
Yes	78 (14.4)	12 (4.9)	**3.27 (1.75,6.13)**	**2.37 (1.07,5.25)**
Alcohol Drinking				
No	273 (50.3)	131 (53.3)	1	1
Yes	270 (49.7)	115 (46.7)	1.13 (0.83,1.52)	0.79 (0.53,1.15)
Cigarette Smoking				
No	493 (90.8)	238 (96.7)	1	1
Yes	50 (9.2)	8 (3.3)	**3.02(1.41,6.47)**	1.37 (0.51,3.73)
Living with steady partner				
No	285 (52.5)	132 (53.7)	1	1
Yes	258 (47.5)	114 (46.3)	1.048 (0.78,1.42)	1.09 (0.60,1.99)
Feeling at risk for HIV				
No	354 (65.2)	167 (67.9)	1	1
Yes	189 (34.8)	79 (32.1)	1.13 (0.82,1.56)	0.93 (0.64,1)
Awareness about HIV care availability				
No	79 (14.5)	38 (15.4)	1	1
Yes	464 (85.5)	208 (84.6)	1.07 (0.71,1.63)	1.08 (0.68,1.72)
Use of traditional treatment				
No	452 (83.2)	212 (86.2)	1	1
Yes	91 (16.8)	34 (13.8)	1.25 (0.82, 1.92)	1.15 (0.71,1.88)
Use of Holly Water				
No	279 (51.4)	119 (48.4)	1	1
Yes	264 (48.6)	127 (51.6)	0.89 (0.66,1.20)	0.72 (0.50,1.04)

Bold: P<0.05

## Discussion

Several previous studies have found high prevalence of late presentation depending on the various definition of late presentation. In this study, late presenter was defined as patients that had been placed on Anti-retroviral therapy in less than 3 months of their first positive HIV test. Accordingly, it was found that late HIV diagnosis occurred in about two third of the study participants (68.82 %). Reports from different Sub-Saharan Africa also found high prevalence of late testers; however, the use of different definitions makes comparisons difficult. For instance, in a study conducted in Nigeria, 50 % of HIV-infected individuals were late diagnosed based on the criteria of CD4 < 200 cells/μL at HIV diagnosis. Similarly, in a peri-urban community near Cape Town, South Africa, 36 % of HIV-infected individuals were late presenter for diagnosis [14].

The main reason for being late in this study was feeling healthy. People often do not attribute their risk behaviors for acquisition of HIV as far as they feel healthy. This has been the case for several previous studies where low perception of individual risk was significantly associated with failure to utilize HIV testing services [9, 15–17]. Although it was not significant, late diagnosis was higher among those that had not felt at risk of HIV-infection than those who did in this study. For many of the study participants, perception of HIV as a highly stigmatizing disease served as a powerful barrier for testing. Other studies from Ethiopia and Sub-Saharan Africa countries also indicated that a perception of HIV as a highly stigmatizing disease was common among people who presented with late-stage disease [9, 18]. It was also found that for some participants in the current study using traditional treatments was the deterrent for having early HIV test. This finding is supported by the study conducted in Uganda that reported those who had received care from non-medical providers were more likely to be diagnosed late [8]. Several factors, in fact, may contribute for the patients to rely on tradition treatments in developing countries such as less coverage of modern health care, perception of the healers as being accessible, nearer, less expensive, and more holistic [19].

Late diagnosis of HIV has important individual, public health and economic consequences. A substantial proportion of people who died from AIDS are late presenters [10]. This study also investigated factors associated with late HIV diagnosis in Southern Tigray, Ethiopia. It was found that use of Khat, ambulatory functional status and bed ridden functional status, being Muslim were found to be the factors that were significantly associated with being late for HIV diagnosis.

Use of khat had an increased odd of being late for diagnosis compared to those who did not chewing khat. Previous studies revealed that substance use such as alcohol and injection drug use implicated with high risk of late diagnosis and care [3, 18, 20]. Khat may produce euphoria, increased confidence, and enhanced alertness [21–23]. This feeling of well being may affect their health seeking behavior and to come late for diagnosis. Moreover, Khat chewers believe that Khat use for relieving stress and pain [24]. A study by Glenice and Ramps showed that khat chewers in Yemen believed Khat was beneficial for mild ailments like headache, fevers and depression [25]. This Use of Khat for relieving pain might have made the participants to disregard HIV related symptoms and pain until they were late in the course of HIV progression.

Bedridden functional status was associated more likely for being late than those in working status in this study. This could indicate the presentation of patients at the health facilities during the advanced stage of disease progression. This trend of testing after the immune system is destroyed and opportunistic diseases are emerged has been the case in several studies elsewhere [6, 18, 26]. Interventions such as encouragement from family, friends, and health care providers for these patients to be tested are needed to circumvent the fear of diagnosis and denial of the likelihood of being infected [2].

Another interesting result of the present study was the association of ambulatory functional status with being late for diagnosis. The possible explanations for this could be not feeling being at risk by the participants and misdiagnosis of HIV infection by the health professionals. The latter reason is well-supported by different studies done elsewhere [2, 26, 27] which also that reported the admission of HIV-infected persons in a variety of health care settings in the years prior to diagnosis. To overcome this matter/issue, health professionals should play important role in recommending HIV test not only in presence of AIDS defining diseases but also for specific HIV indicator conditions [28].

In this study Muslim religion showed a significant association with late HIV diagnosis. This might be explained by fear of stigmatization if they found positive for HIV. A study conducted on religion and HIV diagnosis showed that people who disclosed their HIV status were at risk of isolation from mosques/church as those who are HIV positive may be seen as being punished for sins such as promiscuity, and HIV is considered a ‘curse from God’ [29].

The main limitation of this study could be the design employed which was cross sectional. A cross sectional, in general, might suffer from temporal relationship establishment with some variables and could not provide much more substantial evidence of causality, unlike a longitudinal design.

## Conclusion

The magnitude of late presentation for HIV diagnosis was found to be higher among HIV positive patients in South Tigray Zone, Ethiopia. Feeling healthy, fear of stigma and discrimination and using traditional treatment were the reasons for the participants for being late for HIV diagnosis. Use of Khat, ambulatory and bed ridden functional status, being Muslim were the main predictors of late diagnosis in South Tigray Zone, Ethiopia.
